# Interventional Impacts of Watershed Ecological Compensation on Regional Economic Differences: Evidence from Xin’an River, China

**DOI:** 10.3390/ijerph17176389

**Published:** 2020-09-02

**Authors:** Bing Yu, Linan Chen

**Affiliations:** 1 School of Business, Ningbo University, Ningbo 315211, China; chenlinan271@163.com; 2 Donghai Research Institute, Ningbo University, Ningbo 315211, China

**Keywords:** intervention analysis, Theil index, watershed ecological compensation

## Abstract

Watershed ecological compensation (WEC) is a popular and effective policy instrument for promoting the coordinated development of environment protection and the regional economy in river basin areas. WEC affects the regional economic differences between upstream and downstream regions, as well as between protected areas and areas surrounding upstream regions. Thus, it is necessary to quantify these changes to ensure the balanced development of regions after the implementation of ecological compensation. In the present study, we established two types of Theil indexes for between-group inequalities (T_HH_ and T_HS_) and an intervention analysis model in order to evaluate and predict the effects on regional economic differences caused by WEC in the Xin’an River basin. The results showed that the intervention comprising WEC affected regional economic differences, where the economic gap widened between Huangshan City in the upstream region and Hangzhou City in the downstream region, as well as between Huangshan and its surrounding cities. However, the impacts of the intervention gradually decreased in the later pilot period. Considering the fairness of regional social development, we recommend increasing the compensation for protected areas in order to improve the self-development capacity of upstream regions.

## 1. Introduction

Payment for ecosystem services (PES) is considered one of the best market-based instruments for achieving environmental outcomes, and it has been widely implemented around the world [1,2]. China has also implemented many PES equivalent initiatives during recent decades, and it is often known as ecological compensation (EC) [3]. A successful PES or EC program may pursue ecological goals, but it also needs to facilitate socioeconomic development in the target areas [4]. In particular, watershed ecological compensation (WEC) is used to mediate the ecological and economic conflicts between upstream and downstream areas in a river basin, and thus social equity and economic factors are very important for ensuring the reasonable allocation of responsibilities and obligations in these processes [5]. Nevertheless, the environmental performance of PES or EC has been the main focus in previously reported evaluations [6,7]. Thus, it is necessary to explore the socioeconomic effects of WEC to achieve sustainable watershed development.

Due to the rapid growth in the number and volume of transactions in PES and EC schemes, there is an increasing interest in evaluating the impact of these schemes in China and other countries [8,9]. The importance of evaluations is recognized for guiding policy design and implementation, and many studies have attempted to assess the ecological and socioeconomic impacts of PES and EC programs. In particular, Adhikari and Agrawal [1] analyzed the social and ecological outcomes of 26 PES projects in Asia and Latin America, and found that most had low to medium equity and livelihood outcomes, but they scored better in terms of environmental and ecological sustainability outcomes. Subsequently, Yang and Lu [10] conducted a systematic review of evaluations of EC (PES in China) programs and also found that social equity appeared to be the biggest challenge because none of the main types of EC programs produced highly positive outcomes. These consistent results may validate the argument that ecosystem services are the primary goals of EC or PES projects, and thus the ecological outcomes are the main concern. However, if a PES program aims to be sustainable in the long terms, it needs to satisfy three criteria comprising ecological effectiveness, economic efficiency, and social equality [11,12].

Social equity is considered to be subjective and it cannot be analyzed scientifically [13], but some quantitative comparative analyses have been conducted to explore various issues since 2013, such as the ecological and social effectiveness of programs and tradeoffs [14]. According to previous studies, the social impacts of PES are mainly related to equity, well-being, poverty, and property rights, or they are collectively referred to as livelihoods in some studies [15].

According to previous studies, the socio-economic impacts of PES programs are not consistent across different scales and stakeholders [16,17]. In particular, some studies have confirmed that PES programs can have both positive and negative effects on local households [18]. Moreover, some studies have demonstrated that equity impacts and feedback effects need to be considered at different spatial and temporal scales. Some effects are likely to be considered unfair to local stakeholders, but they might be considered positive at other spatiotemporal scales [19]. Moreover, there are links between the factors that affect both environmental outcomes and welfare [20]. The effectiveness of PES at achieving multiple objectives simultaneously is considered to be debatable [21]. In this context, researchers have started to analyze the social and ecological tradeoffs or synergies related to PES implementations [22]. Unfortunately, few studies have demonstrated the existence of synergies regarding the ecological performance of PES and social outcomes [15,23]. Furthermore, issues have been explored in terms of how policy instruments might interact with targeted social–ecological systems. For example, Sattler et al. [24] discussed when and under what circumstances the implementation of PES schemes would generate benefits, and how these programs could be improved. Pascual et al. [25] showed how the equity impacts of PES can create positive and negative feedback to influence ecological outcomes. Börner et al. [14] highlighted the key contextual and design factors that limit the potential of PES to achieve effective and socially desirable outcomes, such as inadequate or poorly functioning institutional settings. Yang et al. [26] identified the effects of PES programs on socioeconomic outcome based on their influence on different livelihood activities.

Thus, an increasing number of evaluation studies have considered social aspects more deeply by focusing on impacts and their causes. In contrast, the evaluations of EC have lagged behind those of PES, particularly assessments of the social impacts of EC. EC is considered as equivalent to PES or PES-like in many studies, but EC is typically not included in mainstream PES studies [27,28]. WEC has developed rapidly in China and more than 20 provinces have issued WEC policies in recent years. To guide these implementations, two policy documents regarding WEC schemes were issued by the Chinese central government in the past two years. The first document is the “Opinions of CPC Central Committee and the State Council on Establishing a More Effective New Mechanism for Coordinated Regional Development” [29]. The other document is the “Comprehensive Compensation Pilot Plan for Ecological Compensation”, which proposes the promotion of implementations of WEC pilots and conducting evaluations of their performance [30]. Thus, assessing the performance of WEC in upstream and downstream areas of basins from social and economic viewpoints is now a priority.

In the present study, we explored the fairness of social development in the upstream and downstream areas of the Xin’an River basin in China after the implementation of a WEC scheme. Using a Theil index, we calculated and predicted the impacts of the intervention scheme on regional economic differences. The remainder of this paper is organized as follows. In Section 2, we describe the details of the WEC scheme on the Xin’an River. The Theil index and intervention analysis models are introduced in Section 3. The main results are presented in Section 4. We discuss the results and their implications in Section 5. We give our conclusions in Section 6.

## 2. Case Study: WEC Scheme on Xin’an River, China

**Figure 1:**
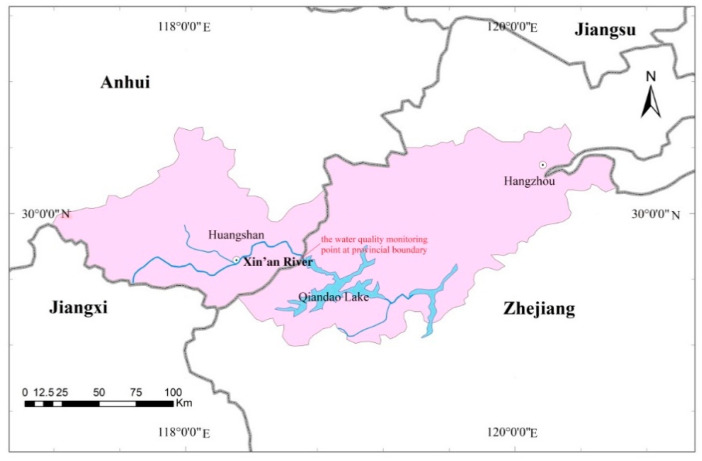
Location of Xin’an River.

The Xin’an River originates in Huangshan City, Anhui Province and flows into Qiandao Lake across the provincial boundary in Hangzhou City, Zhejiang Province (Figure 1). The Xin’an River is an important ecological security barrier and strategic water source for the Yangtze River Delta in China. In the 1990s, the water quality became increasingly eutrophic downstream of Qiandao Lake [31]. The discrepancy also deepened between the need to protect water resources and economic growth in the upstream and downstream regions. In order to address these issues and promote regional sustainable development, the provincial governments of Anhui and Zhejiang signed the “Agreement on Water Environmental Compensation for Xin’an River Basin” in 2012 [31]. This was the first trans-provincial EC pilot in China and the lateral EC mode for a domestic watershed was also created. In this EC pilot, the central government and two provinces agreed to jointly pay for the WEC scheme. Since 2012, this scheme has undergone three phrases with increasing water quality requirements, as shown in Table 1.

According to the news reports, the water quality in the Xin’an River and the eutrophication of the Qiandao Lake have improved significantly after the implementation of that WEC scheme. Specifically, from 2012 to 2017, the water quality across the provincial boundary in Xin’an River had reached a second-class standard of surface water environmental quality, and the nutritional status of Qiandao Lake had changed from mesotrophic to oligotrophic [32]. Moreover, Jing and Zhang [33] confirmed that the ecological compensation in the Xin’an River significantly reduced the intensity of water pollution in Huangshan City and Hangzhou City. However, since 2012, Huangshan has made great efforts to reach the agreed target, where over 12 billion CNY has been invested to prevent pollution and improve the water quality [33]. Despite the increasing ecological benefits for the upstream and downstream regions, the influence of this WEC on the fairness of social and economic development in the upstream region has attracted much concern.

The scope of the WEC scheme mainly covers the whole territory of Huangshan City in the upstream region and Hangzhou City in the downstream region. From the perspective of regional economic differences, the intervention has resulted in two main effects on the upstream region. The first effect is on the economic difference between the upstream area of Huangshan and the downstream area of Hangzhou. The economic level of Huangshan has always been far behind that of Hangzhou because the former is a typical mountainous area whereas the latter is the provincial capital. However, since the implementation of the pilot, the economic development of the upstream region has been severely restricted due to high environmental standards, thereby exacerbating the economic gap between Huangshan and Hangzhou. The second effect is on the economic difference between the city that implemented the WEC and those without the WEC, i.e., Huangshan and the other non-provincial capital cities in the same province. Coordinated regional development demands that the two effects of the intervention cannot be ignored during the WEC process and they need to be studied quantitatively, which was the aim of the present study.

## 3. Methods

### 3.1. Theil Index of Between-Group Inequalities

The Theil index is also known as the Theil entropy, and it was proposed by Theil. It is one of the inequality measures that belongs to the entropy measures from information theory. Theil considered inequality as a by-product of the information content of the structure of the income distribution [34].

The Theil index is used to evaluate and measure the inequality in a given study area. If the area is divided into m groups, the Theil index can be decomposed in two components: the Theil index of within-group inequalities and Theil index of between-group inequalities. The within-group component describes a part of overall inequality that is due to inequality within subgroups, while the between-group component measures the extent of inequality due to the differences in the group mean income [35].

In this study, the Theil index of between-group inequalities is used to assess the regional economic difference between urban groups, and it is calculated as follows:

(1) $$T=\overset{q}{\underset{q=1}{\sum }}y_{q}log\frac{y_{q}}{p_{q}}\;,$$

where T is the Theil index of between-group inequalities, which represents the regional economic difference between groups, n cities in the targeted region are divided into q groups, $y_{q}$ is the ratio of the per capita gross domestic product (GDP) for group q relative to that for the whole region, and $p_{q}$ is the ratio of the population in group q relative to that in the whole region. The T value falls between 0 in the case of perfect equality and $\log\left(\frac{1}{p_{q}}\right)$ for perfect inequality.

According to Equation (1), T_HH_ and T_HS_ indexes were established in this study to represent the regional economic differences between Huangshan and Hangzhou, and between Huangshan and its surrounding areas (other non-provincial capital cities in Anhui Province), respectively. These indexes were used to analyze the regional economic differences and their changing trends after the implementation of the WEC in the Xin’an River basin. The data for population and GDP used in this study were collected from An Hui Statistical Yearbook [36] and Hang Zhou Statistical Yearbook [37] from 2002 to 2019.

### 3.2. Intervention Analysis Model

Intervention analysis was introduced by Box and Tiao [38]. This quantitative method is used to evaluate the effect of an intervention on a time series and it has been widely applied in environmental and economic policy assessments [39,40]. This method is generally used in combination with autoregressive integrated moving average (ARIMA) models. The main aim of this approach is to examine how a time series changes after an intervention when the same ARIMA structure for the series is assumed to hold before and after the intervention [41]. In the present study, the implementation of the WEC in the Xin’an River basin in 2012 was treated as an intervention event and the effects of the WEC on regional economic differences were assessed using this approach. The detailed methods are explained as follows.

An ARIMA(p,d,q) model for T before the intervention was established using time series data $x_{t}\;(t<\;2012)$:

(2) $$\emptyset (B)\Delta^{d}x_{t}=\mu +\theta \left(B\right)\epsilon_{t},$$

where B is the backward shift operator, ∅(B) is the autoregressive (AR) operator, $\Delta^{d}x_{t}$ is the difference in $x_{t}$ after the change in order to making the series stationary, θ(B) is the moving average (MA) operator, and $\epsilon_{t}$ is the white noise at time t.

According to Equation (2), the intervention impact value $Z_{t}$ can be obtained by:

(3) $$Z_{t}=x_{t}-y_{t},$$

where $x_{t}$ comprises the original series values for T (t≥2012) representing the outcome for value T at time t with the intervention, and $y_{t}$ denotes the corresponding predicted values for T (t≥2012) given by the ARIMA model representing the value of T at time t without the intervention. Considering that the impact of the WEC in the Xin’an River basin has strengthened gradually over the long term, the general intervention model for the WEC can be written as follows:

(4) $$Z_{t}=\frac{\omega \left(B\right)}{\delta \left(B\right)}I_{t}^{T}=\frac{\omega }{1-\delta B}S_{t}^{T}\;,$$

where *ω* and *δ* are parameters, and $S_{t}^{T}$ represents the continuous intervention impact given by $S_{t}^{T}=\{\begin{array}{c} 0\;\left(t<2012\right)\\ 1\;\left(t\geq 2012\right) \end{array}$.

The purification series $Y_{t}$ refers to the series that eliminates the influence of the intervention, where it comprises: $Y_{t}=\{\begin{array}{c} x_{t}\;\left(t<2012\right)\\ x_{t}-\frac{\omega }{1-\delta B}\;\left(t\geq 2012\right) \end{array}$. This series can be represented with an improved ARIMA model. Thus, the expression of the total intervention analysis model is given as follows.

(5) $$x_{t}=Y_{t}+\frac{\omega }{1-\delta B}S_{t}^{T}$$

## 4. Results

### 4.1. Regional Economic Differences between Huangshan and Hangzhou

**Figure 2:**
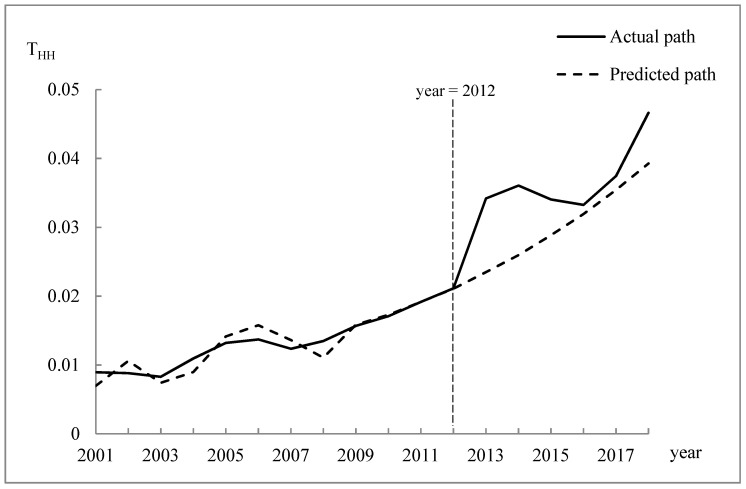
Actual and predicted paths of the T_HH_ index from 2001 to 2018.

The actual changes in the T_HH_ index from 2001 to 2018 were calculated as shown in Figure 2. The actual path in the figure shows that the regional economic difference between Huangshan City and Hangzhou City generally increased, but the upward trend differed significantly before and after 2012. The difference increased more rapidly after 2012, with the largest increase from 2012 to 2013 when the T_HH_ value rose by 61.78%. This change also implies that the WEC pilot implemented in 2012 interfered with the difference in regional economic development between the upstream and downstream regions. In order to evaluate the effects of the intervention over time, we established an ARIMA(1,2,1) model to represent the time series for T_HH_ before the intervention and to generate the series of T_HH_ predictions without the WEC policy in 2012–2018. The predicted counterfactual path of T_HH_ is also shown in Figure 2. The significance test results for the ARIMA model are shown in Table 2.

As shown in Figure 2, the actual value of T_HH_ increased more than the predicted value after 2012, and the fluctuation was more obvious, thereby indicating that the WEC launched in the Xin’an River basin in 2012 increased the regional economic difference between Huangshan City and Hangzhou City. In particular, during 2013, 2014, 2015, 2016, 2017, and 2018, the actual T_HH_ values increased by 45.56%, 38.90%, 18.04%, 4.15%, 5.67%, and 18.72%, respectively, compared with the predictions. Thus, the WEC policy significantly widened the economic gap between the two regions during the first pilot period (2012–2014). However, the difference between the actual value ($x_{t}$) and the predicted value ($y_{t}$) decreased significantly after the second pilot (2015–2017). Therefore, the impact of the WEC policy intervention on the economic difference between Huangshan and Hangzhou gradually decreased but with a rebound in 2018.

According to Equation (4), we measured the estimated intervention effect given by: $Z_{t}=0.055Z_{t-1}+0.006$. The purification series $Y_{t}$ was estimated with the ARIMA(1,2,1) model. The significance test results for this model are also shown in Table 2. Thus, the total intervention analysis model was obtained as: $x_{t}=Y_{t}+\frac{0.006}{1-0.055B}S_{t}^{T}$. Using this model, we predicted the actual values of the T_HH_ index ($x_{t}$) and the intervention impact value ($Z_{t})$ from 2019 to 2021, as shown in Table 3. These results demonstrated that the regional economic difference between Huangshan and Hangzhou still widened further, but the impact of the WEC policy intervention decreased.

### 4.2. Regional Economic Difference between Huangshan and Surrounding Areas

In addition to the vertical difference between the upstream and downstream regions, we also measured the horizontal difference based on comparisons with other cities in the same province. We selected 14 non-provincial capital cities in Anhui province with the same administrative level as Huangshan City, and the average economic level of these cities was used for the analysis. In addition, some of the prefecture-level cities in Anhui Province were established at different times, so the average per capita GDP and average population in each year were calculated based on the number of cities that became prefecture-level cities in that year in order to ensure a fair comparison.

**Figure 3:**
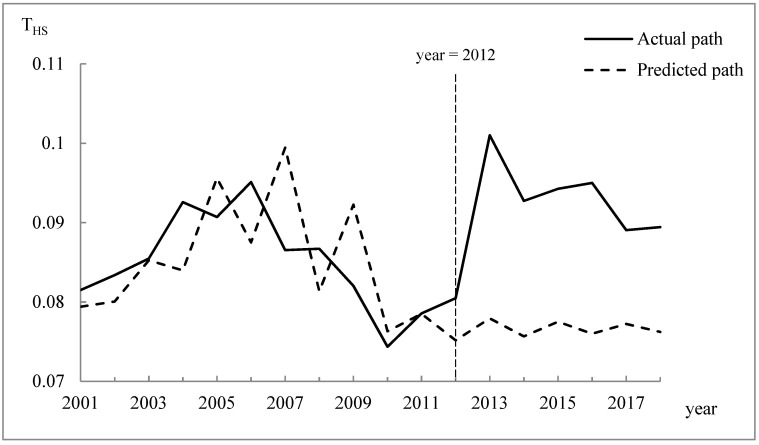
Actual and predicted paths of the T_HS_ index from 2001 to 2018.

The changes in the actual T_HS_ index from 2001 to 2018 are shown in Figure 3. The results demonstrated that the regional economic difference between Huangshan City and other prefecture-level cities in Anhui Province varied, but the magnitude of this fluctuation differed significantly before and after 2012. The regional economic difference exhibited a clear upward trend after 2012, where the increase was the largest from 2012 to 2013, reaching 25.52%. Therefore, the implementation of the intervention policy in 2012 affected the regional economic difference between Huangshan City and the surrounding areas. To evaluate the effects of the intervention over time, we established an ARIMA(1,1,1) model to represent the time series for T_HS_ before the intervention and to generate the predicted series of T_HS_ without the WEC policy in 2012–2018. The predicted counterfactual path of T_HS_ is also shown in Figure 3. The significance test results for the ARIMA model are shown in Table 4.

By comparing the two paths in Figure 3, we can see that the actual values of T_HS_ were higher than the predicted values after 2012 and the fluctuations were greater. In particular, the actual values of T_HS_ in 2013, 2014, 2015, 2016, 2017, and 2018 increased by 29.62%, 22.56%, 21.62%, 25.01%, 15.31%, and 17.33%, respectively, compared with the predicted values. Therefore, the WEC launched in the Xin’an River basin in 2012 significantly increased the regional economic difference between Huangshan and other prefecture-level cities in Anhui Province, but the influence of the intervention decreased.

We also measured the estimated intervention effect given by: $Z_{t}=-0.332Z_{t-1}+0.022$. In addition, the purification series $Y_{t}$ was estimated with the ARIMA(1,1,3) model. The significance test results for this model are also shown in Table 4. Thus, the total intervention analysis model was obtained as: $x_{t}=Y_{t}+\frac{0.022}{1+0.332B}S_{t}^{T}$. This model was used to predict the actual values of the T_HS_ index ($x_{t}$) and the intervention impact value ($Z_{t})$ from 2019 to 2021, as shown in Table 5. The results showed that the regional economic difference between Huangshan and its surrounding cities continued to increase, but the impact of the WEC policy intervention gradually decreased.

## 5. Discussion

### 5.1. Comparisons with Other Studies

In recent years, several studies have investigated the economic effects of WEC policies on the EC areas in China. For example, Yang and Fu [42] theoretically analyzed the effect of WEC on the economic growth in an upstream region based on an endogenous growth model. They found that simply using the environmental quality to set the standard for compensation was not entirely effective for the upstream region. The design of EC should satisfy the need to consider the dynamic relationships among pollution regulation, ecological payment, and economic growth. Subsequently, an empirical study was conducted by Zhang et al. [43] to evaluate the effect of the WEC in the Xin’an River basin on economic growth in Huangshan City from 2012 to 2016. They found that ecological protection reduced the per capita GDP in Huangshan City by a level of 2.93%. These studies demonstrated that the current WEC design hindered the economic development of the upstream compensated area, which is consistent with the conclusion obtained in the present study.

Compared with previous investigations, the present study provides novel insights into the influence of the WEC on regional economic differences in the Xin’an River basin. First, we showed that the WEC affected the economic growth in the upstream region, thereby aggravating the discrepancy between the development of the upstream and downstream regions. Therefore, the socioeconomic impacts need to be analyzed further from the perspective of the relationship between the two regions. Second, the effect of the intervention on the regional economic differences varied during the different periods of the WEC program. Thus, the dynamic predictions obtained in this study may help to improve the follow-up mechanism.

### 5.2. Implications for WEC

According to intervention analysis and the predictions obtained in this study, the WEC in Xin’an River basin may have increased the regional economic differences over a certain time, thereby resulting in imbalanced regional development. Therefore, it is necessary to take appropriate measures to improve the horizontal EC mechanism by balancing the interests of the upstream and downstream stakeholders and coordinating regional development.

The results obtained in this study confirmed that the EC resulted in the economic development of Huangshan lagging behind. According to field research, Huangshan’s investment in water environmental protection and ecological restoration far exceeded the compensation funds it received under the agreement. Furthermore, a large number of companies have been closed down and transferred, and many projects could not be developed due to the limitations imposed by the water environment goals. In summary, the current high “input” and low “output” situation have resulted in backward economic development, and thus the following improvements need to be implemented.

First, the compensation standard needs to be improved. The essence of compensation is to internalize the externality of ecological protection in the basin, and to coordinate the relationship between environmental and economic interests for protectors and beneficiaries.

From the perspective of protectors, their internalization requirement due to the WEC is the cost they have paid to protect the ecological environment, including direct costs and opportunity costs. From the perspective of beneficiaries, their internalization requirement due to the WEC is the ecological benefit they enjoy, which is provided by the protectors. However, the existing compensation funds can only pay for the treatment and protection of environmental pollution, and they cannot compensate for the opportunity costs of those people whose interests are damaged as a consequence of the ecological protection process. Therefore, we can state that the current implementation only provided “partial compensation” and it failed to fully internalize the positive externalities of ecological protection.

The people in the upstream region need a certain amount of time to transform their economic behavior. In particular, during the initial stage of compensation, adequate compensation is an important foundation of ecological protection but also important support for the development of a new type of economy. According to our results, the upstream region was clearly still at a stage where economic development was hindered. Therefore, it is necessary to increase the compensation funds and expand the scope to reduce the pressure on environmental protection in the upstream region and increase the capacity for local economic development.

Second, various compensation methods need to be employed to improve the economic development capacity in the upstream area. The ultimate goal of EC is to coordinate ecological and economic development. Therefore, in practice, the EC should also include different stages that consider the initial compensation for income losses to later compensation for lost development. In the initial stage, monetary compensations, namely, fiscal transfers and lateral payments are more recommended. These methods mainly rely on the government, with the features of simple and efficient, and can compensate for the income losses of the upstream in time. In the later stages, the compensation goal is to enhance the upstream economic development capacity. The non-monetary compensation methods, such as counterpart cooperation, industrial transfer, talent training, and joint construction of industrial parks, are highly preferred for improving upstream development [44]. Through these ways, the upper and lower regions of the basin could establish a robust compensation relationship, while the government and market could find ways to work together to improve the efficiency of the compensation system.

The Xin’an River WEC has been piloted for three rounds, and it is timed to the stage of compensation for lost development. The high-quality environment of Huangshan City should become a new local economic growth point. First, the ecological system should be made the basis of the economy by developing and using natural environmental resources as economic resources. For example, an ecological product trading market in the basin should be established by the governments that can reflect the ecological value of the water environment, instead of simply treating the water environment as a basis for rewards and punishments. In addition, the economy should be made ecological by developing industries that are conducive to the ecological environment. In this regard, the assistance of the beneficiary area is required. For example, the downstream can transfer some high-tech industrial projects to the upstream. They can cooperate in green technology innovation, eco-tourism joint development, exhibition economy, etc.

## 6. Conclusions

The WEC intervention scheme in the Xin’an River basin affected the regional economic differences. The implementation of the EC widened the economic gap between the compensated area (upstream of Huangshan City) and the beneficiary area (downstream of Hangzhou City), as well as widening the economic gap between the compensated area and the surrounding prefecture-level cities. The impact of the intervention decreased gradually with the continued implementation of the EC, but with a rebound in 2018. Empirical results showed that the Theil index of between-group inequalities in Huangshan City and Hangzhou City (T_HH_) increased by about 40% annually during the first pilot period of the WEC in the Xin’an River basin (2012–2014), and by about 9% annually during the second pilot period (2015–2017), but by about 19% in 2018. These results demonstrate that the economic development of Huangshan City was severely affected by the impact of the EC intervention in the initial stage. Subsequently, the economic development of Huangshan City was restored to a certain extent, and the discrepancy between the upstream and downstream areas gradually decreased. However, the decreasing trend was unstable, and an increase has appeared in the first year of the third pilot. In addition, the Theil index of between-group inequalities for Huangshan City and its surrounding cities (T_HS_) also increased at a variable rate during the WEC process in the Xin’an River basin, with an annual increase of about 20% but a downward trend. Thus, due to the impact of the WEC intervention in the Xin’an River basin, the economic gap between Huangshan City and its surrounding areas increased significantly during the first pilot period but decreased slightly during the second pilot period. According to the predictions obtained by the intervention analysis model, the impact of the WEC intervention on the regional economic differences between Huangshan and Hangzhou, as well as between Huangshan and its surrounding cities decreased further from 2019 to 2021. Therefore, the EC mechanism should be improved to ensure fair regional social development and to promote the sustainable development of the whole basin.

## Figures and Tables

**Table 1:** Main contents of the three-round pilot agreement on the Xin’an River watershed ecological compensation scheme.

Pilot	Period	Compensation Basis	Compensation Funds ^1^
1st Round	2012–2014	$P=K_{0}\times \overset{4}{\underset{i=1}{\sum }}K_{i}\frac{C_{i}}{C_{i0}}$ P is the compensation index; K_0_ is the water quality stability coefficient, which takes different values in each pilot period; K_i_ is the indicator weight coefficient, which takes different values in each pilot period; C_i_ is the annual concentration value of indicator i; C_i0_ is the basic limit set for indicator i during each pilot period; i denotes water environment indicators, including the permanganate index, ammonia nitrogen, total phosphorus, and total nitrogen	300 million CNY/year from the central government; 100 million CNY/year from Anhui provincial government; 100 million CNY/year from Zhejiang provincial government
2nd Round	2015–2017		900 million CNY from the central government; 200 million CNY/year from Anhui provincial government; 200 million CNY/year from Zhejiang provincial government
3rd Round	2018–2020		200 million CNY/year from Anhui provincial government; 200 million CNY/year from Zhejiang provincial government

^1^ The compensation funds contributed by the provincial government are determined according to the *p*-value. According to the agreement, when the water quality across the provincial boundary satisfies the specific objective (*p*-value), Zhejiang will compensate Anhui; otherwise, Anhui will compensate Zhejiang.

**Table 2:** Significance tests for the ARIMA model for T_HH_.

Model		Coefficient	Standard Error	*p*-Value
$x_{t}$	AR(1)	−0.645	0.213	0.007
	MA(1)	0.999	0.096	0.000
$Y_{t}$	AR(1)	−0.633	0.158	0.001
	MA(1)	0.999	0.058	0.000

**Table 3:** Predictions of T_HH_ ($x_{t}$) and the intervention impact ($Z_{t})$ from 2019 to 2021 in the Xin’an River watershed ecological compensation (WEC).

Index	2019	2020	2021
$x_{t}$	0.052	0.053	0.057
$Z_{t}$	0.008	0.005	0.003

**Table 4:** Significance tests for the ARIMA model for T_HS_.

Model		Coefficient	Standard Error	*p*-Value
$x_{t}$	AR(1)	−0.817	0.057	0.000
	MA(1)	0.999	0.095	0.000
$Y_{t}$	AR(1)	−0.470	0.160	0.006
	MA(2)	0.331	0.147	0.033
	MA(3)	0.818	0.139	0.000

**Table 5:** Predictions of T_HS_ ($x_{t}$) and the intervention impact ($Z_{t})$ from 2019 to 2021 in the Xin’an River WEC.

Index	2019	2020	2021
$x_{t}$	0.094	0.093	0.092
$Z_{t}$	0.018	0.016	0.016
